# Joint Trends in Flood Magnitudes and Spatial Extents Across Europe

**DOI:** 10.1029/2020GL087464

**Published:** 2020-04-01

**Authors:** Matthias Kemter, Bruno Merz, Norbert Marwan, Sergiy Vorogushyn, Günter Blöschl

**Affiliations:** ^1^ Institute of Environmental Science and Geography Potsdam University Potsdam Germany; ^2^ Helmholtz Centre Potsdam, GFZ German Research Centre for Geosciences Potsdam Germany; ^3^ Potsdam Institute for Climate Impact Research Potsdam Germany; ^4^ Institute of Hydraulic Engineering and Water Resources Management Technical University Vienna Vienna Austria

**Keywords:** flood, synchrony, magnitude, climate change, classification, spatial statistics

## Abstract

The magnitudes of river floods in Europe have been observed to change, but their alignment with changes in the spatial coverage or extent of individual floods has not been clear. We analyze flood magnitudes and extents for 3,872 hydrometric stations across Europe over the past five decades and classify each flood based on antecedent weather conditions. We find positive correlations between flood magnitudes and extents for 95% of the stations. In central Europe and the British Isles, the association of increasing trends in magnitudes and extents is due to a magnitude‐extent correlation of precipitation and soil moisture along with a shift in the flood generating processes. The alignment of trends in flood magnitudes and extents highlights the increasing importance of transnational flood risk management.

## Key Points

- Flood magnitudes and extents are correlated across Europe
- Both have increased in central Europe and the British Isles but decreased in Eastern Europe
- These trends are driven by regional changes in flood generation

## 1 Introduction

River floods are among the most harmful natural hazards worldwide, and their damages are expected to increase further as a consequence of climate change, population and economic growth, and rising economic interdependence (Dottori et al., 2018; Field et al., 2012; Kundzewicz et al., 2014; UNDRR, 2019). In Europe, trends in flood magnitudes have been identified (Blöschl et al., 2019; Jongman et al., 2014; Mangini et al., 2018). These trends vary in space because of differences in the flood generating processes. For example, increasing autumn and winter rainfall has resulted in increasing floods in Northwestern Europe, while decreasing snowmelt has led to decreasing floods in Eastern Europe in the past five decades (Blöschl et al., 2017; Mangini et al., 2018). If a flood event covers a large region, emergency response, disaster recovery, and the insurance industry may be overtaxed, as resources and funds need to be provided at many locations at the same time (Jongman et al., 2014). In Europe, the flood extent, that is, the area or distance over which flooding occurs simultaneously, has been found to change (Berghuijs et al., 2019), but the alignment of these changes with changes in the flood magnitudes has not been studied. An alignment of flood magnitude and flood extent trends has the potential of increasing the flood risk beyond the effects of the individual trends. If there are clear physical causes, the alignment may translate into the future.

## 2 Materials and Methods

### 2.1 Data

We use a flood data set consisting of the timing and magnitude of annual maximum discharge for 5245 hydrometric stations in Europe (Blöschl et al., 2019). We choose a timeframe from 1960–2010 to keep the number of available stations for each year relatively constant over time and select only those stations with at least 30 years of data. The selection resulted in a total of 3,872 stations with catchment sizes ranging from 1 to 800,000 km^2^. The median catchment size is 312 km^2^.

In order to examine the process controls on floods, we use reanalysis data (Primo et al., 2019) with a spatial resolution of 0.11 × 0.11°. The variables are precipitation, snowfall, soil moisture (46 cm depth), soil pore space, snowmelt, convective available potential energy (CAPE), and convective inhibition (CIN). The original temporal resolution is 1 hr (precipitation and snowfall), 3 hr (CAPE and CIN), and 1 day (soil moisture and snowmelt). We aggregate all variables to daily totals. These data are used for two analyses: (i) pixel‐based magnitude‐extent correlations and (ii) catchment based identification of flood generation processes. For the latter we derive catchment boundaries, using the CCM (Vogt et al., 2007) and MERIT Hydro (Yamazaki et al., 2019) data sets to calculate catchment average time series of these variables. The daily time series are calculated by weighted averages of the pixels at each time step, where the weight of each pixel is set according to the fraction of its area covered by the catchment area.

### 2.2 Flood Synchrony Scale

We quantify the spatial extent of flood events by the flood synchrony scale (Berghuijs, Allen, et al., 2019). It is defined as the maximum distance from a station within which at least 50% of the stations have the annual maximum flood discharge at the same time as the reference station. We allow for a time delay ∆t of ±7 days in order to account for the travel time of weather patterns to move across Europe and flood routing in the river system. Therefore, the flood synchrony scale (FS) of a station *i* in year *j* is defined as follows:

(1) $$\mathrm{FS}\left(i,j\right)=\max\left\{d\;\left[f\left(d\right)>0.5\right]\right\}$$

where *f* is the fraction of stations within distance *d* where the annual maxima occurred within the allowed time delay *∆t*:

(2) $$\;f\left(d\right)=\frac{1}{n\left(d\right)}\sum_{i=1}^{n\left(d\right)}\left(t_{\mathrm{ref}}-∆t<t_{i}<t_{\mathrm{ref}}+∆t\right)$$

Here *n*(*d*) is the number of stations within distance *d* from the reference station, *t*_*ref*_ is the day of the flood at the reference station, and *t*_*i*_ is the day of the flood at the other stations. We estimate trends of the flood synchrony scale for each station using the Theil‐Sen slope estimator (Sen, 1968). Trends are averaged following 30,000 random station resampling iterations, to minimize the effect of heterogeneous station density (Figure S1 in the supporting information). We then interpolate the trends spatially using the autoKrige function of the R automap package (Hiemstra et al., 2009) to obtain regional trends.

### 2.3 Magnitude Extent Correlation

We estimate the Spearman rank correlation coefficients between the annual series of flood magnitude and flood extent in terms of the flood synchrony scale (equation 1) for each station. We test the significance of the correlation at the 5% level, adjusting the False Discovery Rate of multiple hypothesis testing using the Benjamini‐Hochberg correction (Benjamini & Hochberg, 1995).

Additionally, we evaluate the analogous correlations for precipitation, soil moisture and snowmelt. In this case, we use the gridded data set and calculate a time series of extent for each pixel by the same method as for the flood peaks. For precipitation, soil moisture and snowmelt a day is considered an event if it exceeds the 99% quantile. We compare pixels on the same day (∆*t* = 0) because no river routing is involved. Furthermore, we repeat the analysis for measured satellite and station based precipitation data using PERSIANN (Ashouri et al., 2015) and ECA&D data (Klein Tank et al., 2002), respectively. Again, we evaluate Spearman rank correlation coefficients and apply a Benjamini‐Hochberg correction for the significance tests.

### 2.4 Flood Classification

We classify the total of about 174,000 flood peaks by their dominant flood generating processes. Flood generation can be dominated by the hydrometeorological forcing (rainfall and snowmelt) as well as by the antecedent catchment state (soil moisture and snow cover). We therefore consider the following flood generating processes: convective precipitation, stratiform rainfall, soil moisture excess, snowmelt, and rain‐on‐snow. We use the catchment average time series of the climate variables for the classification along with a simple decision tree (Figure S2). As a first step, we subtract snowfall from precipitation to calculate rainfall. We estimate the concentration time *t*_*c*_ of each catchment by *t*_*c*_ = *αA* ^*β*^, where *α* = 0.1 and *β* = 0.3 with *t*_*c*_ in units days and *A* in units square kilometers (Corradini et al., 1995; Robinson & Snapalan, 1997). For 3,191 stations (82%) *t*_*c*_ = 1 day and for only 39 stations (1%) *t*_*c*_ ≥ 4 days. We consider the climate variables on the day of the flood peak as well as *t*_*c*_ days prior to it for the classification. If snowmelt was greater than rainfall, we classify the flood generating process as snowmelt. If snowmelt was less than rainfall but most of the catchment (>66% of the area) was covered by any amount of snow, we classify the flood as rain‐on‐snow. If this was not the case, we check for convective conditions in the catchment. We detect these by using thresholds (Findell & Eltahir, 2003) for CAPE (>400) and CIN (<5) and assign convective rainfall as a generating process, if at least 25% of the catchment area had convective conditions on any day during *t*_*c*_. If none of the above conditions applied, we check whether the soil water content exceeded 70% of the available pore space on the day prior to *t*_*c*_. If this was the case, we classify the flood as soil moisture excess related. All floods that did not meet any of the above criteria are considered to be mainly caused by stratiform rainfall. While floods are often caused by the interplay of different parameters, we only classify them by the dominant process for simplicity and clarity. The thresholds for CAPE and CIN we use here do not guarantee convective conditions, as high vertical wind shear values can also be necessary (Gilleland et al., 2016). As this parameter was not available to us, it is likely that the classification overestimates the frequency of convective rain.

While the classification concept used here is simple, it allows for a fast automatic classification of a large number of flood peaks based on widely available climate data. It considers flood generation processes beyond the timing of the flood within the year and therefore may provide more detailed information than timing‐based classifications (Mediero et al., 2015; Tarasova et al., 2019). For instance, it never classifies a flood as snowmelt related unless there was a substantial amount of snow present in the catchment, whereas timing‐based methods do not make this distinction. To check the plausibility of the classification results we examine the temporal distribution of the flood generation processes across the year.

For each catchment, we estimate the relative frequencies of flood peaks caused by a process (termed relevance), assigning 1 if a flood peak was associated with a process in a given year and 0 if it was not. We calculate their trends in the period 1960–2010 by the Theil‐Sen estimator and spatially interpolate both the relevance and its trend by kriging using autoKrige as before. Finally, we estimate the regional diversity ( *D*_*j*_) of flood generation processes by

(3) $$D_{j}=1-\mathrm{var}\left(f_{m,j}\right)$$

where *j* is the year, *m* is the flood generation process (1, …, 5), and *f*_*m*, *j*_ is the relative frequency of process *m* in year *j*. We calculate *D*_*j*_ for two regions, one in Western Europe where flood magnitudes have increased and one in Eastern Europe where they have decreased (Blöschl et al., 2019). For comparison, we estimate the spatial variance *T*_*j*_ of the flood dates by the circular variance:

(4) $$T_{j}=1-\sqrt{{\bar{\cos\left(\theta_{i,j}\right)}}^{2}+{\bar{\sin\left(\theta_{i,j}\right)}}^{2}}$$

where *θ* is the flood date converted into an angle between 0 (1 January) and 2*π* (31 December), *i* is the station, and *j* is the year (Table S1).

## 3 Results

**Figure 1:**
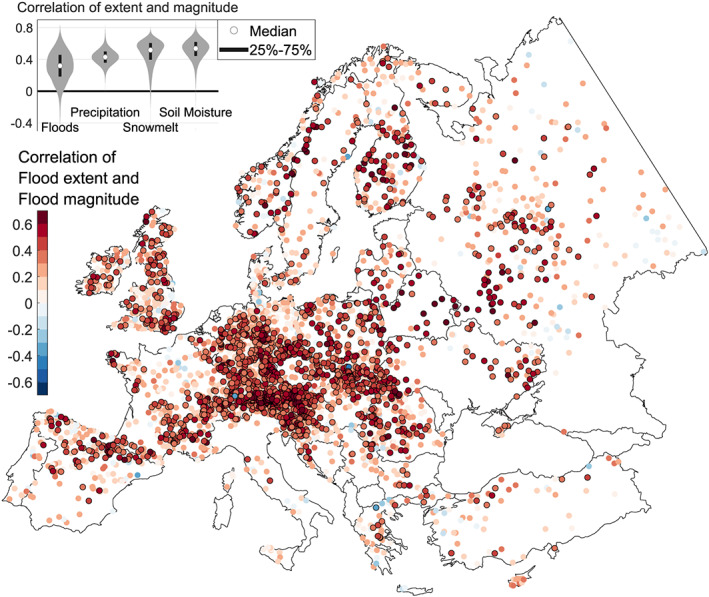
Spearman Rank Correlation of flood extent and flood magnitude. Annual series 1960–2010 (*N* = 3872). Positive correlations are shown in red and negative in blue. Stations with significant correlations are indicated by black edges. The consistent positive correlation implies that high magnitude floods tend to be associated with large spatial extents all over Europe. The inset in the upper left corner shows the distribution of this correlation for floods as well as for three flood controls.

Our data show significant positive correlations between annual flood magnitudes and their flood synchrony scale for 46.5% (*N* = 1,802) of the stations (Figure 1). We find significant negative correlations for only 0.1% (*N* = 4) of the stations. A total of 95.2% (*N* = 3,685) and 4.8% (*N* = 187) of the stations exhibit positive and negative correlations, respectively. The positive correlations are highly consistent across Europe. This is related to the similar correlations of three controls on floods (Figures S3–S5). Specifically, the average correlations between magnitudes and the corresponding synchrony scales of precipitation, soil moisture excess, and snowmelt are 0.44, 0.54, and 0.52, respectively (Figure 1 inset), while the average correlation for floods is 0.32. The presence of magnitude‐extent relationships of the controls suggests that they propagate to the floods. The spatial extent of localized convective storms has been previously shown to increase with precipitation magnitudes (Lochbihler et al., 2017; Molnar et al., 2015), and this analysis suggests that this is also the case at the regional scale. For a given weather system velocity, large‐scale precipitation events tend to produce longer rainfall than short‐scale events, which appears to translate into higher daily precipitation (Skøien et al., 2003). Similarly, the moisture content of soils close to saturation, which often occur in winter, tends to be more homogeneous over larger regions than that of soils if average moisture content (Pachepsky et al., 2003). Snowmelt (Figure S5) also shows mostly positive correlations, because snowmelt will occur over large regions during events with high temperatures. The correlation values of precipitation extents and magnitudes for the PERSIANN and ECA&D data are similar to those presented above, which suggests that the correlations are not an artifact of the reanalysis data set.

**Figure 2:**
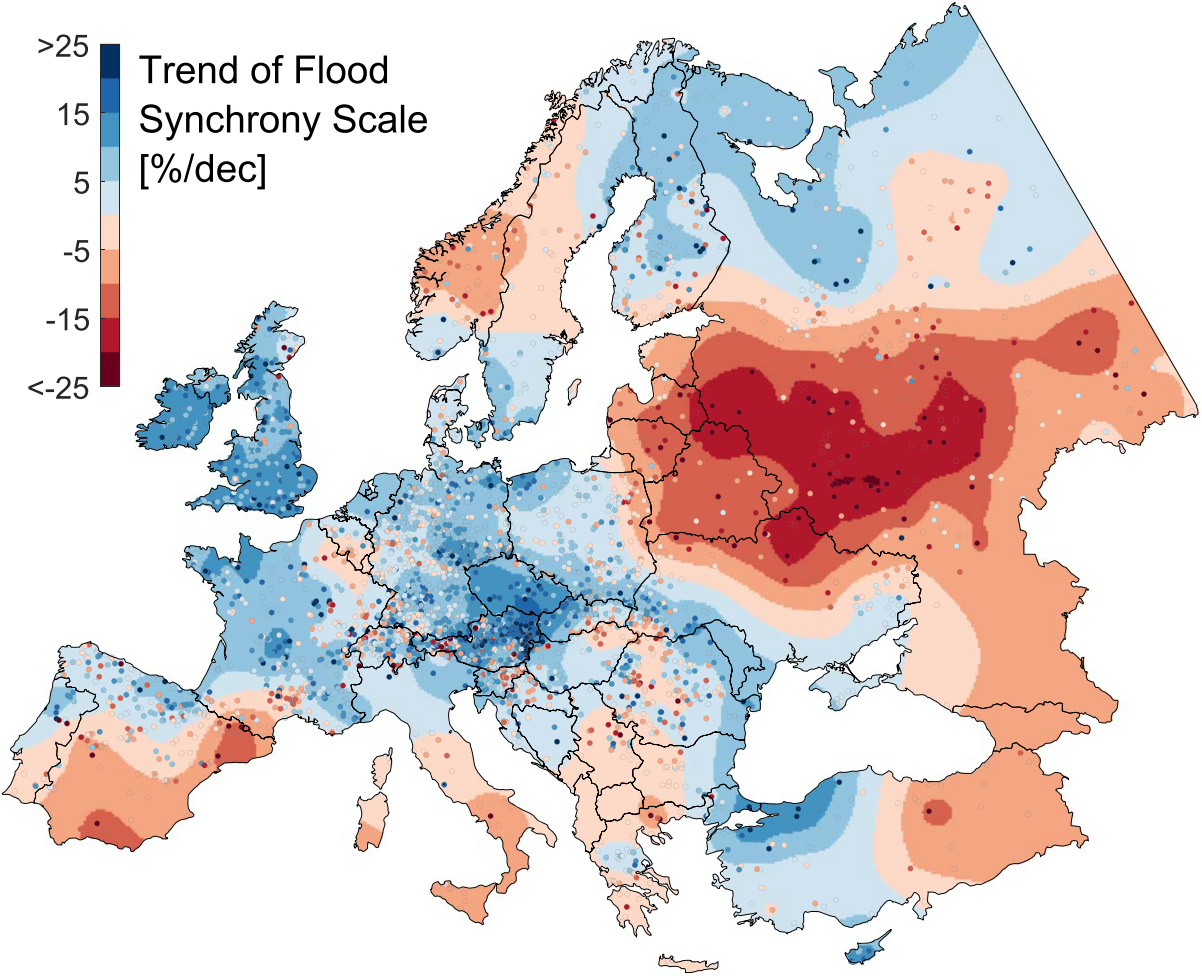
Observed trends of flood extent in Europe, 1960–2010. Blue indicates increasing flood synchrony scales, and red denotes decreasing flood synchrony scales (in percent change of the mean scale per decade). The station‐based trends (shown as dots) are spatially interpolated to obtain the regional trends (background color).

The flood extent, in terms of the flood synchrony scale, averages 140 km across Europe but varies regionally (Berghuijs, Allen, et al., 2019). The trends in the flood extent show clear spatial patterns (Figure 2). Relative to the mean flood extent over 1960–2010, regional trends range from an increase of +19% to a decrease of −20% per decade (Figure 2). On the British Isles, in central Europe, and at the Atlantic coast, the flood synchrony scales have increased by about 9% per decade. In Eastern Europe, the flood synchrony scales have decreased by about −11%. The spatial patterns of the trends in extent are closely aligned with those in flood magnitudes, that is, increasing trends in Northwestern and parts of central Europe and mostly decreasing trends in the rest of the continent (Figure S6). The correlation between the trends of flood magnitude and trends of flood extent of individual stations across Europe is 0.31 (*N* = 3,872), but the regional trends are more correlated (*r* = 0.59), as some of the estimation uncertainty is removed (Blöschl et al., 2019).

**Figure 3:**
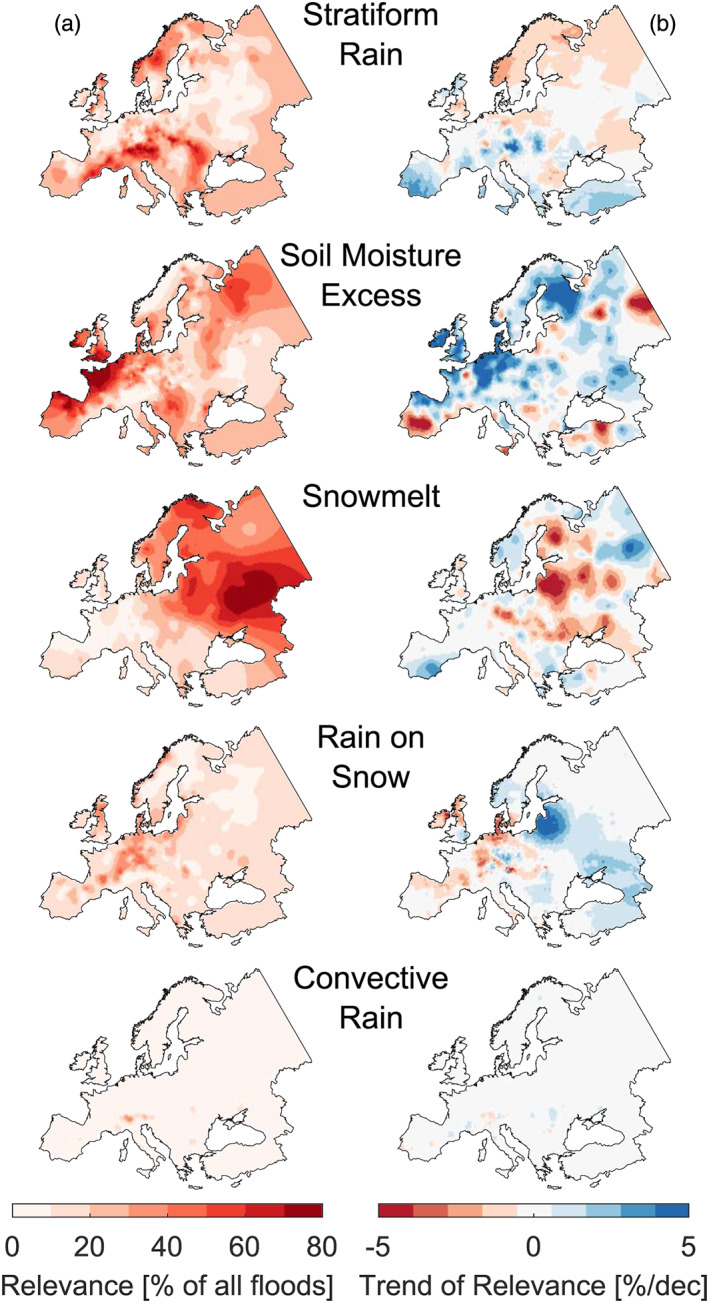
Relevance of flood generating processes and corresponding trends. (a) Relevance of each process quantified by the relative frequency of floods caused by that process in the period 1960–2010. (b) Trends of the annual relevance (in percent change of the mean relevance per decade). Only stations with at least five floods caused by the respective process are considered in the trend analysis.

Our classification shows that the relevance of the flood generation processes varies across Europe (Figure 3a). Stratiform rainfall is particularly important in the Alps and the Carpathians, soil moisture excess in the Atlantic climate of Western Europe, and snowmelt in the north and east of Europe. Rain‐on‐snow has some relevance in the midmountain ranges of central Europe. The spatial patterns of stratiform rainfall, soil moisture excess, and snowmelt controls on floods are in agreement with a previous study (Berghuijs et al., 2019). The low frequency of convective floods in this classification is because such floods usually occur in catchments of a few square kilometers (Merz & Blöschl, 2003). The median catchment size of the flood data set used here is 312 km^2^, so most convective floods are not captured in the data (Blöschl et al., 2019). As would be expected, soil moisture and rain‐on‐snow generated floods mainly occur in winter while convective and stratiform rainfall floods mainly occur in summer (Figure S7).

In the various regions of Europe, the relevance of flood generation processes has shifted during 1960–2010 (Figure 3b). The relevance of stratiform rainfall has decreased in the north but increased along the Mediterranean coast. The relevance of soil moisture excess has increased on the British Isles and in central and Northern Europe. The relevance of snowmelt has decreased in Eastern Europe, where it is the most important process. The relevance of rain‐on‐snow has decreased in Western Europe but increased in parts of Eastern Europe.

The process analysis (Figures 3, S3–S5, and Tables S1 and S2) explains why the flood extents have changed in Europe and why these extent changes are aligned with changes in the flood magnitudes. The increased flood extent in central Europe and the British Isles is related to the increases in precipitation and soil moisture (Blöschl et al., 2019) along with the significant correlation between the magnitudes and extents of these two variables (Figures S3 and S4), which propagates to the floods. Additionally, there is a shift toward soil moisture excess related floods that possess larger flood synchrony scales (Table 1).

**Table 1:** Statistics of the Flood Generating Processes

Process	Stratiform rainfall	Soil moisture excess	Snowmelt	Rain on snow	Convective rainfall
Overall relevance (% of all floods)	31.0	26.0	21.5	18.1	3.4
Average trend of relevance (% per decade)	0.49	1.55	−1.65	−0.41	−0.06
Average flood synchrony scale (distance as % of station average)	92.0^*^	113.2^*^	93.6^*^	107.2^*^	73.9^*^
Average flood magnitude (% of station average)	100.7^**^	103.2^*^	97.0^*^	98.0^*^	98.8^**^

*Note*. Floods that are generated by different processes have significantly different spatial extents (synchrony scales). The large‐extent soil moisture related floods have increased in frequency. The average scaled flood magnitudes are almost independent of the flood generation process

* Synchrony scales and flood magnitudes significantly different from the overall mean (*p* = 0.001).

** No significance.

In Eastern Europe, the opposite is the case. The decreasing flood extent is related to decreasing snowmelt along with a significant correlation between the magnitude and extent of snowmelt in this part of Europe (Figure S5). Additionally, there is a shift toward a larger diversity of flood generation processes, measured by their variance, which increases by 3.2% per decade (Table S1), thus reducing the flood synchrony scale. This change is aligned with an increasing variance of the timing of the annual floods by 7.3% per decade, as other processes than snowmelt increase in frequency (Table S1). Finally, the shift from snowmelt toward rain‐on‐snow and soil moisture excess related floods increases the frequency of floods with smaller flood synchrony scales in this region (Table S2).

Our findings are consistent with observed shifts toward later floods in the North Sea region and parts of the Mediterranean coast and shifts toward earlier floods at the Atlantic coast and the continental northeast (Blöschl et al., 2017) associated with changes in the timing of snowmelt, winter storms, and soil moisture excess maxima.

## 4 Conclusions

We present a clear alignment of flood magnitudes and extents, both in terms of absolute values and trends. Additionally, we determine climatic magnitude‐extent correlations and shifts in flood generation that explain these trends. The processes explaining the observed alignment of increasing trends in parts of Europe and the consistency with climate projections (Thober et al., 2018) emphasize the role of climate change in flood changes and the possibility that these changes may persist into the future. For example, in central Europe and the British Isles, flood extents of about 43 km have increased to about 110 km during 1960–2010. If these trends continue, the alignment of magnitude and extent trends may pose more serious challenges to flood management than expected, highlighting the importance of transnational cooperation in emergency response, disaster recovery, and flood risk management.

## Supporting information



Supporting Information S1
