# Supplementary material for: Joint Trends in Flood Magnitudes and Spatial Extents Across Europe
Source: Geophys Res Lett. 2020 Apr 1;47(7):e2020GL087464. doi: 10.1029/2020GL087464 (PMC8651004; doi:10.1029/2020GL087464)
Supplement: Supplementary file 1 — Supporting Information S1 [file GRL-47-e2020GL087464-s001.docx]

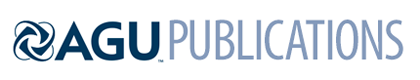


*Geophysical Research Letters*

Supporting Information for

Joint Trends in Flood Magnitudes and Spatial Extents across Europe

Matthias Kemter^1,2,3,*^, Bruno Merz^1,2^, Norbert Marwan^3^, Sergiy Vorogushyn^2^, Günter Blöschl^4^

1 Institute of Environmental Science and Geography, Potsdam University, Am Neuen Palais 10, 14469 Potsdam, Germany.

2 Helmholtz Centre Potsdam, GFZ German Research Centre for Geosciences, Telegrafenberg, 14473 Potsdam, Germany.

3 Potsdam Institute for Climate Impact Research, Telegrafenberg, 14473 Potsdam, Germany.

4 Institute of Hydraulic Engineering and Water Resources Management, Technical University Vienna, Karlsplatz 13, 1040 Vienna, Austria.

Corresponding author: Matthias Kemter (kemter@uni-potsdam.de)

**Contents of this file**

Figures S1 to S7

Tables S1 to S2

**Introduction**

This document contains seven figures and two tables to support the findings of the paper. All of them are referred to in the paper.


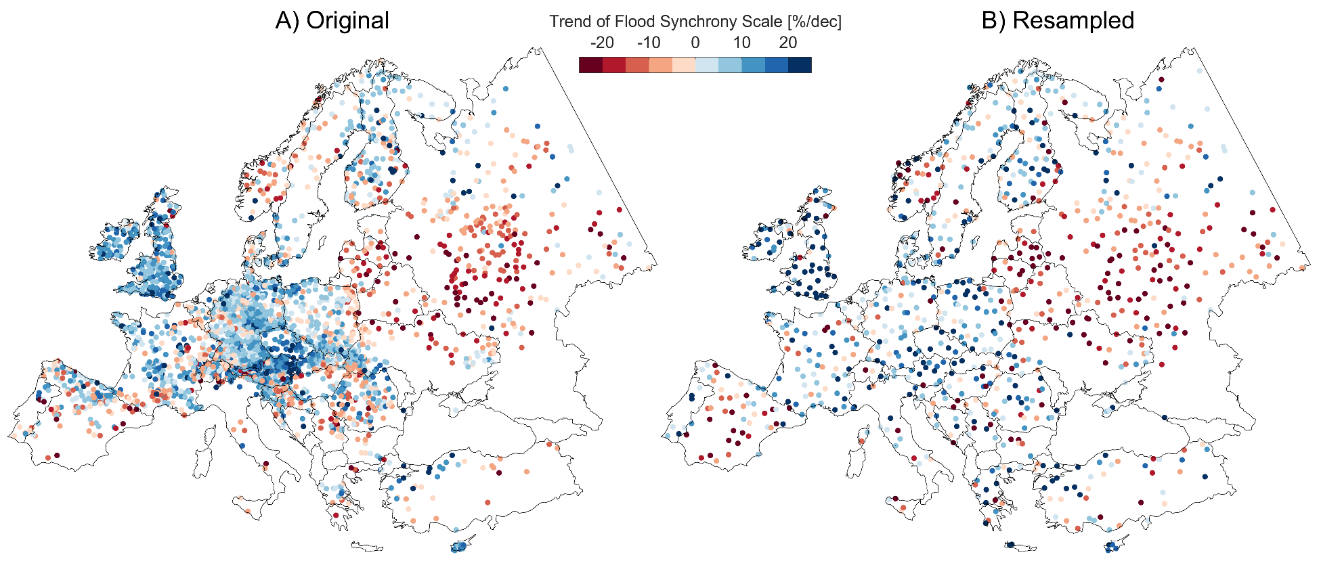
 Figure S1. Sensitivity of trend in flood synchrony scale to station density. In the complete dataset, (A) central Europe and the UK are much more densely filled with stations than the rest of Europe. By randomly sampling one station per 1x1° grid cell, we achieve a more homogeneous density of stations (B). The regional patterns in flood extent trends do not change as a result of the resampling.

**Figure S2.** Classification workflow of the flood generating processes. The time window is set according to the catchment area. Each flood peak is classified according to the catchment conditions by five consecutive binary decisions.


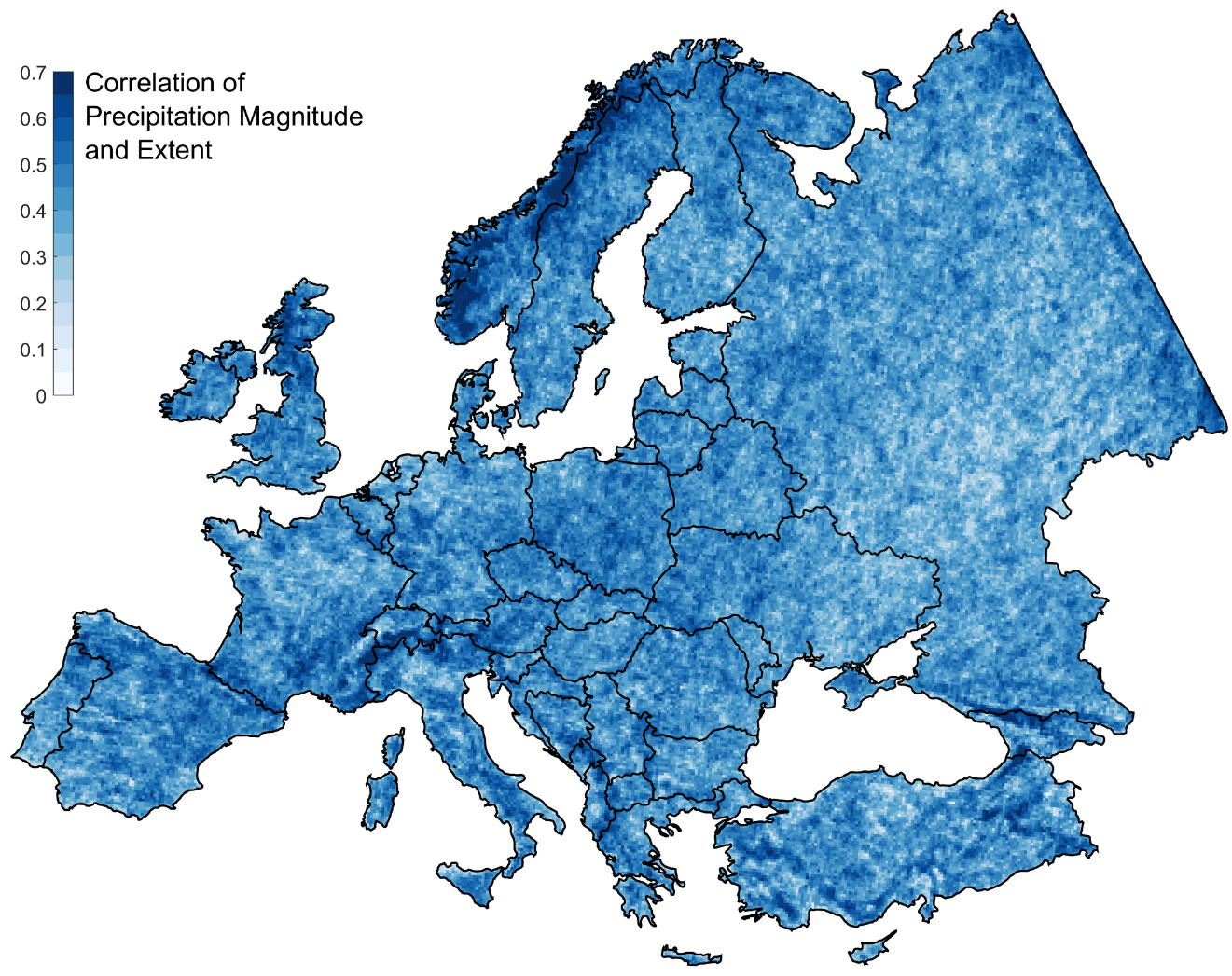


**Figure S3.** Correlations between annual series of the magnitude of 99th percentile precipitation and associated extents. We find positive correlation in virtually all of Europe. For 99.7% of pixels, the correlation is significant at the 95% level.


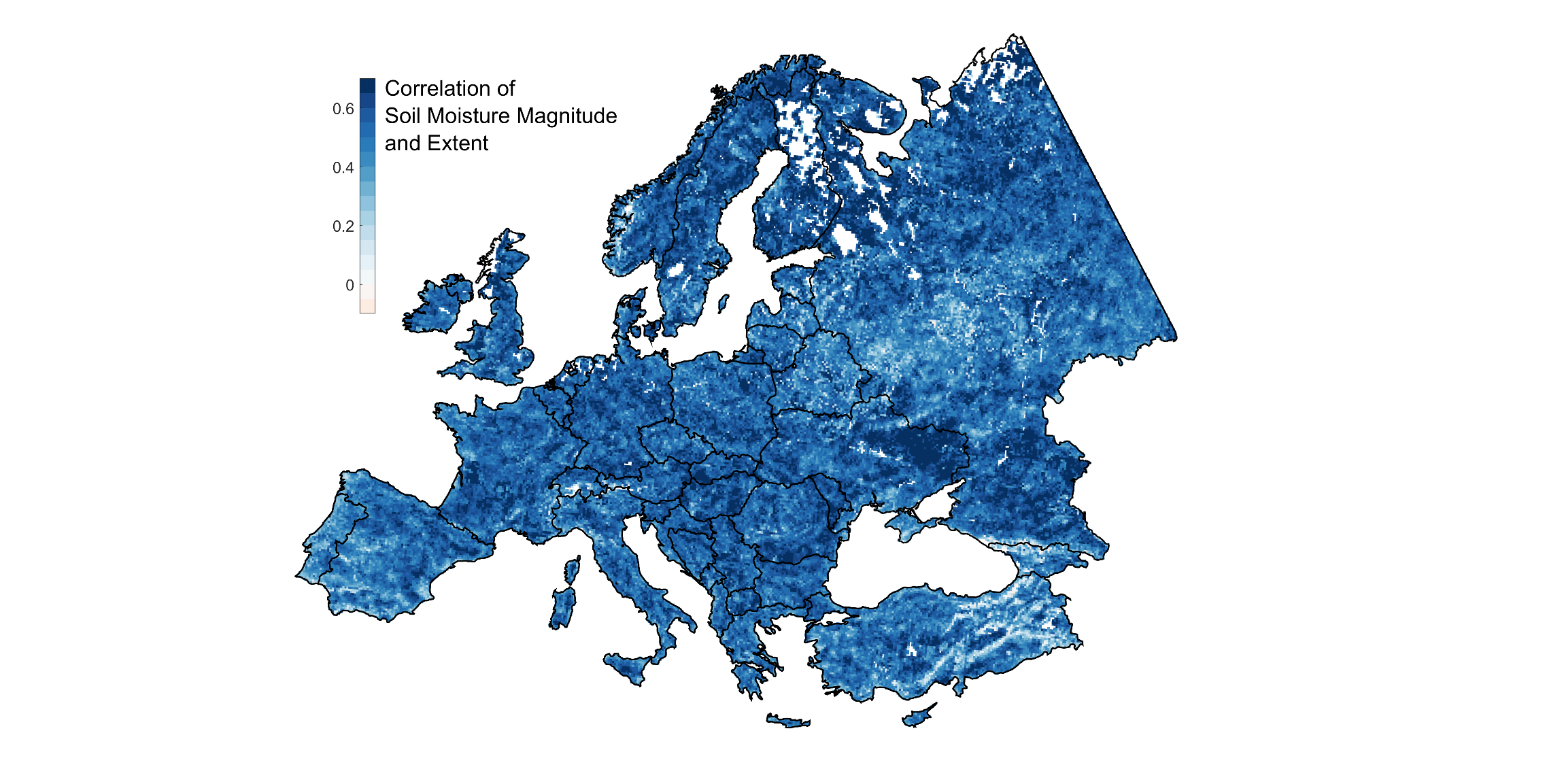


**Figure S4.** Correlations between annual series of the magnitude of 99th percentile soil moisture and associated extents. We find positive correlation in most parts of Europe. For 99.1% of pixels, the correlation is significant at the 95% level.


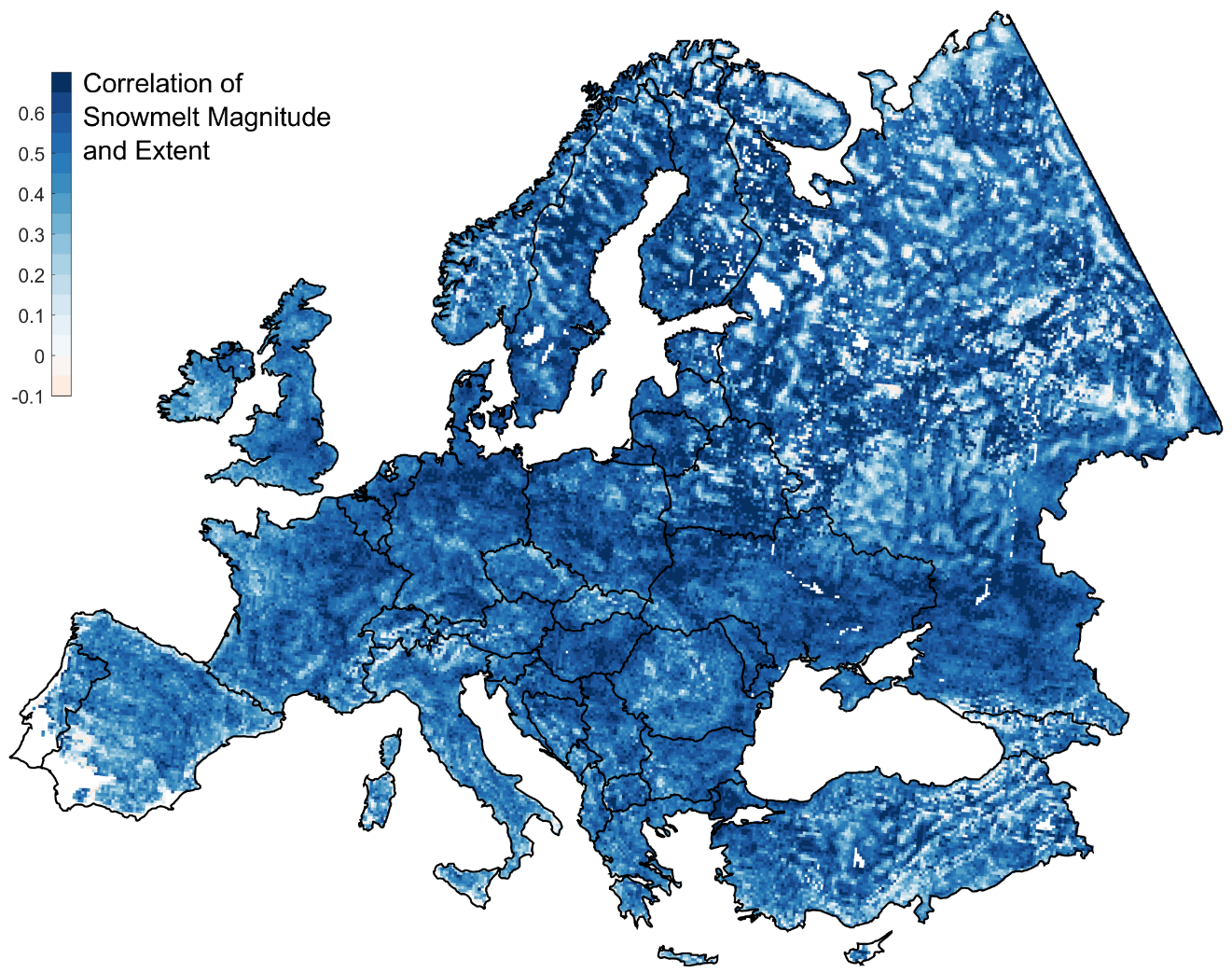


**Figure S5.** Correlation between annual series of the magnitude of 99th percentile snowmelt and associated extent. Only pixels with at least 10 snowmelt events in the period 1960-2010 were considered. We find positive correlation in most parts of Europe. For 95.3% of pixels, the correlation is significant at the 95% level.


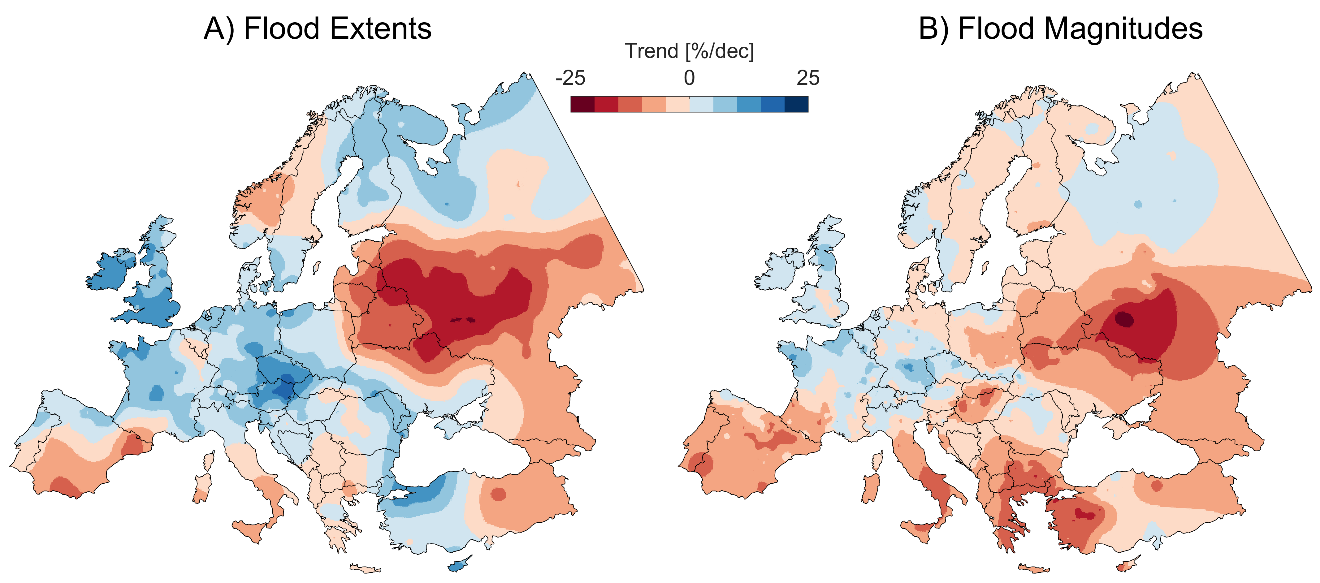


**Figure S6.** (A) Regional trends in flood extent and (B) Regional trends in flood discharges in Europe (1960–2010) (Blöschl et al., 2019). Blue indicates increasing extents and discharges and red denotes decreasing extents and discharges (in percent change of the mean per decade).


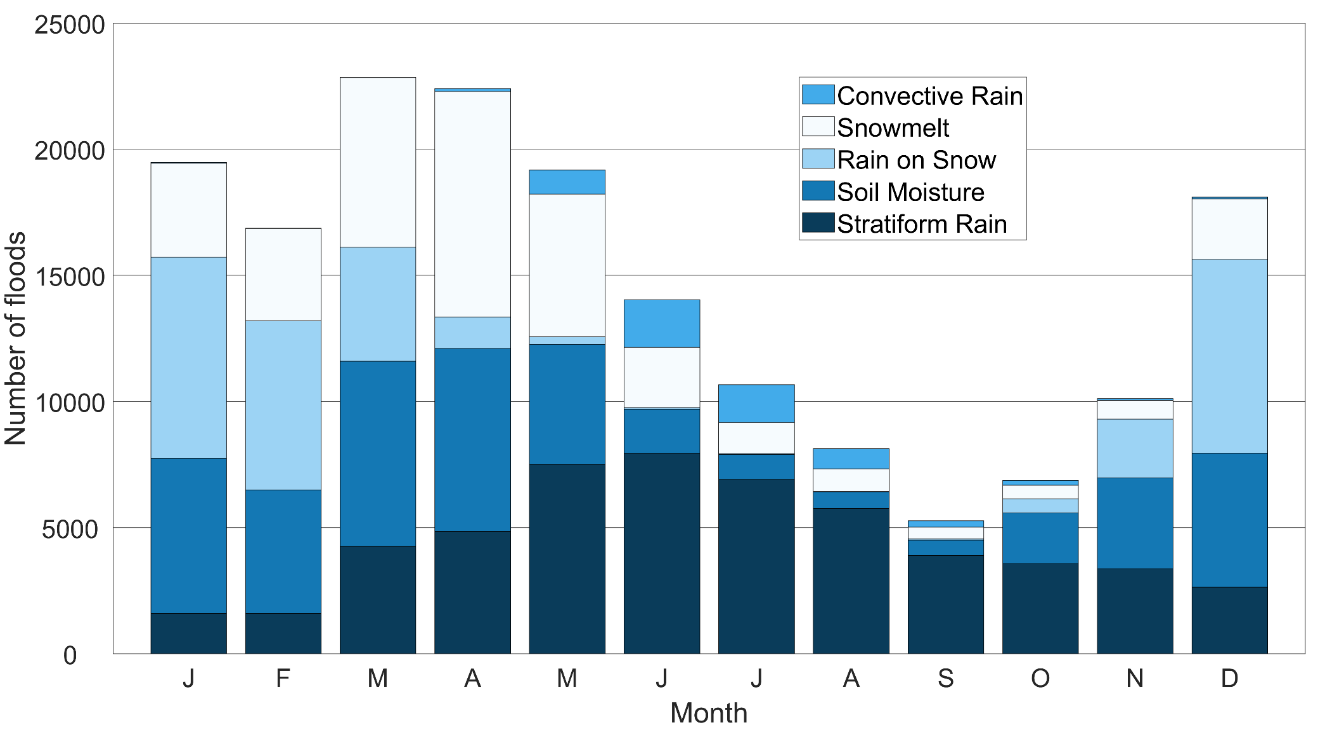


**Figure S7.** Distribution of the flood generation processes across the year. All processes occur predominantly in the expected seasons (rain-on-snow in winter, convective rainfall in summer, etc.).

| **Region** | **Europe** | **Region West** | **Region East** |
| --- | --- | --- | --- |
| **Number of stations** | 3872 | 2394 | 308 |
| **Flood synchrony scale trend**  **(% per decade) [station based]** | 6.8 | 8.5 | -10.7 |
| **Percentage of stations with significant synchrony scale trends**  **(%, positive/negative)** | 37.8/15.9 | 49.2/6.7 | 4.2/62 |
| **Trend of flood synchrony scale**  **(% per decade) [interpolated pixels]** | -1.2 | 5.9 | -8.5 |
| **Flood magnitude trend**  **(% per decade) [station based]** | 0.2 | 1.9 | -6.9 |
| **Percentage of stations with significant flood magnitude trends (%, positive/negative)** | 8.7/11.9 | 11.7/4.7 | 0.9/36.7 |
| **Trend of flood date variance**  **(% of average per decade)** | 0.43^†^ | 0.75^†^ | 7.3* |
| **Trend of flood type diversity (% of average per decade)** | -0.35^†^ | -0.79* | 3.2* |

**Table S1.** Trends of flood synchrony scales (extents) and magnitudes, as well as trends in the variance of flood dates and the diversity of flood generation processes in Europe and in two regions. Region West consists of Austria, Belgium, Czech Republic, Denmark, France, Germany, Ireland, Luxembourg, the Netherlands, Poland, Slovakia, Switzerland and the United Kingdom. Region East consists of Belarus, Estonia, Latvia, Lithuania, Russia (south of 60°N) and the Ukraine (east of 27.5°E). Significance of the trends was tested by a Mann Kendall test (p=0.05). Asterisk (*) indicates significant trends, dagger (†) indicates no significance. In the East, the timing of flood occurrence gets more variable and the floods generation processes get more diverse, while in the West these changes are very small.

| **Average flood synchrony scale for each generation process**  **(% of station average)** | **Europe** | **Region West** | **Region East** |
| --- | --- | --- | --- |
| **Stratiform Rain** | 92.0* | 90.2* | 77.9* |
| **Snowmelt** | 93.6* | 85.3* | 108.4* |
| **Soil Moisture** | 113.2* | 114.9* | 102.3^†^ |
| **Rain on Snow** | 107.2* | 112.2* | 73.4* |
| **Convective Rain** | 73.9* | 72.3* | 32.1* |

**Table S2.** Average synchrony scales (extents) for floods with different generation processes in Europe and in two regions (see caption Table S1). Asterisk (*) indicates synchrony scales significantly different (p=0.001) from the overall mean of all generation processes in the respective region, dagger (†) indicates no significance.
